# STRATEGIES FOR MAINTAINING FIDELITY AND SUSTAINABILITY IN EVIDENCE-BASED PROGRAM DELIVERY

**DOI:** 10.1093/geroni/igad104.1018

**Published:** 2023-12-21

**Authors:** Kate Gordon, Carey Wexler Sherman, Kenneth Hepburn

**Affiliations:** Splaine Consulting, Silver Spring, Maryland, United States; Savvy Systems, LLC, Minneapolis, Minnesota, United States; Emory University, Atlanta, Georgia, United States

## Abstract

Federal agencies and advisory councils emphasize the need to expand delivery of evidence-based programs (EBPs) to meet the needs of the increasing millions of family members and friends providing long-term, complex care to persons living with dementias in the U.S. (Alzheimer’s Association, 2022). Achieving lasting value and impact of such implementations requires systematic and replicable programmatic fidelity and sustainability to ensure caregivers’ broad access and positive outcomes, and the significant investments of providers, stakeholders, and funders are maintained. Unfortunately, a paucity of reported evidence on such strategies continues to limit the full benefit of most intervention efforts. (Hailemariam, M., Bustos, T., Montgomery, B. et al. 2019). To inform the larger RO1 effort described in the previous two papers, we recruited nine organizations from diverse regions that deliver Savvy Caregiver® to discern the key strategies employed to ensure program fidelity and sustainability. Semi-structured interviews were conducted with organization directors (n=9) and Savvy coordinators and group leaders (n=23). Thematic content analyses of transcribed transcripts yielded salient findings on fidelity best practices, including: Assuring EBP coherence with an organization’s mission; proactively integrating fidelity into interventionists’ training; and integration of fidelity monitoring strategies. Regarding structural practices supporting sustainability, findings included: Engaging leadership buy-in of Savvy as an anchor program; establishing succession plans for seamless program delivery; creation of local collaborative partnerships for long-term recruitment or in-kind contributions; establishment of infrastructure to secure ongoing state and federal financial support. Findings highlight effective, accessible, and achievable strategies for ensuring long-term benefits for all EBP implementation efforts.

